# A prospective randomized-controlled non-blinded comparative study of the JAK inhibitor (baricitinib) with TNF-α inhibitors and conventional DMARDs in a sample of Egyptian rheumatoid arthritis patients

**DOI:** 10.1007/s10067-024-07194-x

**Published:** 2024-10-31

**Authors:** Esraa M. Mahmoud, Abdullah Radwan, Sahar A. Elsayed

**Affiliations:** Rheumatology and Rehabilitation Department, Faculty of Medicine, Sohag University, Sohag, Egypt

**Keywords:** Baricitinib, TNF-α inhibitors, CDMARDs

## Abstract

To evaluate the efficacy of baricitinib compared to TNF-α Inhibitors and conventional DMARDs (cDMARDs) in patients with RA. Our study included 334 RA patients classified into 3 groups: the first receiving baricitinib, the second receiving TNF-α Inhibitors, and the third receiving cDMARDs. Patients were evaluated at baseline, week 12, and week 24 using TJC, SJC, VAS, DAS28, CDAI, and HAQ-DI. Larsen score was measured at baseline and 24 weeks. The response to therapy was assessed at weeks 12 and 24 using ACR 20, ACR 50, and ACR 70 response criteria. Emerging treatment side effects were monitored. Patients receiving baricitinib showed significant improvement regarding all outcome measures at weeks 12 and 24. In addition, baricitinib was comparable to TNF Inhibitors in all outcome measures except the ACR 70 at week 12, which was higher in the baricitinib group. Furthermore, baricitinib group showed significantly better outcome measures and response to therapy in comparison to cDMARDs group. The most common side effects in the baricitinib group were infection, GIT, and CVS complications. The most common side effects in the TNF inhibitors group were infection and skin complications. The cDMARDs had the least side effects, mostly GIT complications. Baricitinib is an effective drug for treating RA refractory to cDMARDs, improving disease activity measures and functional status and reducing the progression of structural joint damage. It has a comparable efficacy and safety profile to TNF Inhibitors. Multicenter studies are recommended to support our results.

**Table:** 

**Key Points** • *Baricitinib is an effective therapeutic choice for rheumatoid arthritis refractory to cDMARDs.* • *Patients treated with baricitinib showed improvement in all outcome measures and functional status.* • *Bricitinib delayed the progression of radiographic joint damage more effectively than cDMARDs.* • *The efficacy and safety of baricitinib for treating rheumatoid arthritis is comparable to that of TNF inhibitors.*

**Key Points**

• *Baricitinib is an effective therapeutic choice for rheumatoid arthritis refractory to cDMARDs.*

• *Patients treated with baricitinib showed improvement in all outcome measures and functional status.*

• *Bricitinib delayed the progression of radiographic joint damage more effectively than cDMARDs.*

• *The efficacy and safety of baricitinib for treating rheumatoid arthritis is comparable to that of TNF inhibitors.*

## Introduction

Rheumatoid arthritis is the most common form of inflammatory arthritis [1]. In addition to joint inflammation, destruction, and loss of function, RA can cause several extra-articular manifestations [2], together with significant comorbidity associated with an increased cardiovascular risk and dyslipidemia [3]. Fortunately, early recognition of the disease and the considerable improvement seen over the past decade regarding RA management can lead to faster achievement of treatment goals, better health outcomes, and preservation of joint function [4].

The recent management guidelines for RA recommended the initiation of Methotrexate monotherapy in all cases with moderately to severely active disease [5, 6]. Despite being highly effective, many cases fail to achieve clinical disease remission with either MTX monotherapy or in combination with other cDMARDs [7, 8]. The continuous effort to understand the pathogenesis of RA has yielded remarkable insights towards developing new therapeutic agents that can help achieve disease control and overcome cDMARDs failure or intolerability [9].

TNF-α plays a vital role in RA pathogenesis. It has been detected in the synovial fluid and synovium [10]. TNF-α is essential for the pathogenesis of inflammatory osteolysis, recruitment of osteoclasts, and promotion of bone destruction. So, it plays a vital role in bone degradation [11]. Adding TNF-α inhibitors to the RA treatment protocol has dramatically improved the treatment outcomes, with significantly better regain of physical function, quality of life, and prevention of joint damage. Sustained control of the manifestations of RA has also become more achievable [12]. However, the high cost, parenteral route of administration, as well as the remaining sizeable number of patients with poor disease control or drug intolerability, all have raised the need for an effective, orally available, less costly alternative. JAK inhibitors are the most recently developed class of DMARDs used to treat RA. Their effect depends on the role played by Janus Kinases (JAK) and the signal transducer and activator of transcription (STAT) in cytokine signaling in RA [13]. JAK inhibitors are approved for patients with moderate or highly active RA and those with inadequate response to cDMARDs [14]**.** They represent the first effective oral treatment option for cases with refractory RA. Their effect is comparable to the existing biological DMARDs [15]**.** This study was carried out to assess the efficacy of the JAK inhibitor (baricitinib) in patients who had no or poor disease control despite the use of cDMARDs and to compare it with TNF inhibitors and cDMARDs.

## Patients and methods

This study included adult RA patients who attended the rheumatology department of the university hospital from October 2022 to December 2023. Our patients were diagnosed according to ACR/EULAR 2010 classification criteria [16]. Patients with autoimmune diseases other than RA, previous treatment with any tsDMARD or bDMARD, contraindications for TNF inhibitors and baricitinib, patients unable to provide written informed consent, or not using contraceptives were excluded from our study.

### Sample size calculation

According to Lauper et al. [17], who found that JAK inhibitors were less often discontinued than TNF inhibitors for ineffectiveness (adjusted Hazard Ratio (aHR) 0.75, 95% CI, 0.67 to 0.83), at alpha error 0.05, power of the study 80%, JAK inhibitor to TNF inhibitor ratio 2:1 and according to the following formula:

$$\frac{{({Z}_{1-\beta }+{Z}_{1-a})}^{2}}{pA\times pB\times \text{log}{(HR)}^{2}}$$

Z_1-α_ = constant that reflects alpha error 0.05 and 95% confidence interval, Z_1-β_ constant that reflects the power of the study (80%), *pA* is the assumed hazard ratio (HR) in the reference*. pB* = *1- pA.*

### The estimated sample size was

**Fig. 1:**
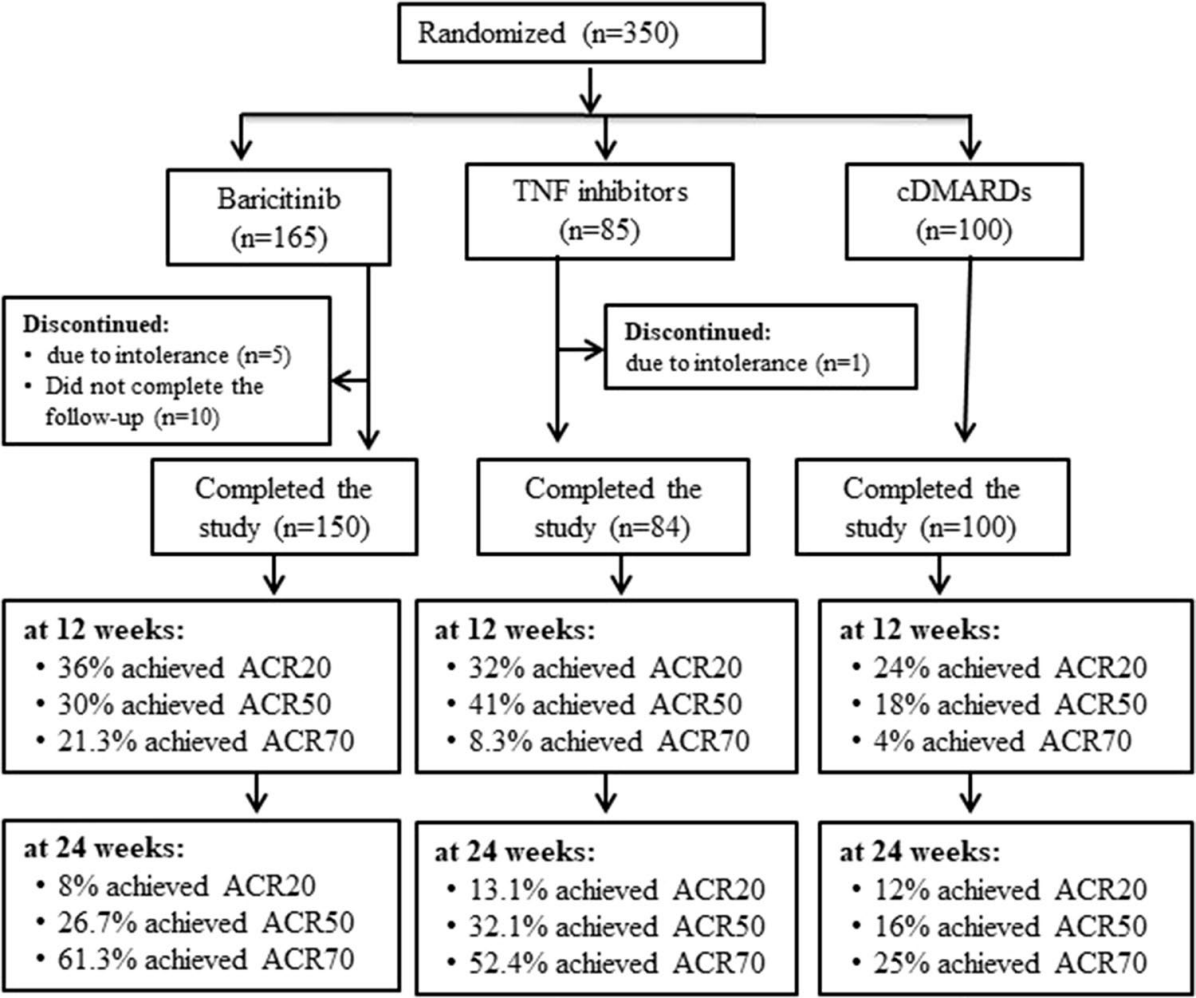
Study flow diagram

The JAK inhibitor group = 150 patients and the TNF inhibitors group = 75 patients, after the addition of 10%–15% of the sample to compensate for missing patients during the follow-up, and the cDMARDs receiving patients were added as a control group. The sample size is demonstrated in Fig. 1

### Patient’s classification

Three hundred fifty patients with moderate to highly active disease were enrolled in our study. Sixteen patients withdrew from the study, from which ten patients did not complete the follow-up period, six patients stopped the medications due to drug intolerance, and the total number of patients who completed the study was 334 patients [296 females (88.62%) and 38 males (11.38%)]. We categorized the patients into three groups: Group 1: patients receiving the JAK Inhibitor baricitinib at 4 mg daily, included 150 patients, and represented 44.91% of the study population. Group 2: patients receiving TNF inhibitors (Etanercept at 50 mg/week, Golimumab at 50 mg/month, and Adalimumab at 40 mg/2 weeks) included 84 patients and represented 25.15% of the study population. Group 3: patients receiving cDMARDs included 100 patients and represented 29.94% of the study population as shown in Fig. 1.

### Data collection

Demographic data, physical and rheumatological examinations were done, and the therapeutic program was recorded. Laboratory investigations included the ESR, CRP, CBC, RF, ACPA, ALT, AST, serum creatinine, and urea. Evaluation of the patients was done at enrollment (baseline). Follow-up was done at weeks 12 and 24.

Treatment response and patients outcome were assessed using:

- **Tender joint count (TJC)** at initial presentation (baseline) and weeks 12 and 24.
- **Swollen joint count (SJC)** at initial presentation and weeks 12 and 24.
- **Visual analog scale (VAS)** at the initial presentation, weeks 12 and 24, the patients expressed their degree of pain over the last week by placing a mark between "no pain" (left end, 0 cm) and excruciating pain (right end, 10 cm) [18].

#### Disease activity score for 28 joints (DAS28)

DAS28 was calculated using the formula of the number of tender and swollen joints, the VAS, and the ESR. Patients were evaluated at the initial presentation, weeks 12 and 24**.** Remission means DAS28 ≤ 2.6, low disease activity means 2.6 < DAS28 ≤ 3.2, moderate disease activity means 3.2 < DAS28 ≤ 5.1, and high disease activity means 5.1 < DAS28 [19].

#### Clinical disease activity index (CDAI)

It uses a formula with the same parameters as DAS28, but it doesn’t require the level of acute phase reactants. Patients were evaluated at the initial presentation, 12 and 24 weeks**.** CDAI ≤ 2.8 stands for remission, 2.8 < CDAI ≤ 10 stands for low disease activity, 10 < CDAI ≤ 22 stands for moderate disease activity, and CDAI > 22 stands for high disease activity [20].

#### Functional status assessment by health assessment questionnaire disability index (HAQ-DI)

Patients were evaluated at baseline, 12 and 24 weeks from starting therapy. Eight questions were used: eating, dressing, rising, walking, hygiene, grip, reach, and usual activities. Each question is answered on a 4-level impairment scale ranging from zero to three: Zero = no difficulty, one = some difficulty, two = much difficulty, and three = inability to do [21].

#### American college of rheumatology 20%, 50%, and 70% response criteria (ACR 20, ACR50, and ACR70)

It measures 20%, 50%, and 70% improvement (respectively) in tender or swollen joint counts in addition to 20%, 50%, and 70% improvement in three of the five ACR core set measures: pain, patient and physician global assessments, disability, and an acute phase reactant [22, 23].

#### Larsen score

X-rays of both hands and feet were done, and structural joint damage was measured by the modified Larsen scoring system at the initial presentation and 24 weeks, with a maximum score of 160 [24].

#### Side effects of medications

Any emerging symptoms or signs suggesting infection or other adverse effects were evaluated at the initial presentation, 12 and 24 weeks.

### Statistical analysis

Data were analyzed using the version 24 statistical package (IBM-SPSS). Data were represented as numbers, percentages, mean, and standard deviation (SD). For quantitative data, the Student's t-test was used to compare the means between two groups, the one-way-ANOVA test was used to compare the means between three or more groups, and the paired samples t-test was used to compare the means of two quantitative variables in one group. We used the chi-square test to compare the percentages of qualitative variables between groups. We used the McNemar test to compare the percentages of two qualitative variables in one group.

## Results

Three hundred thirty-four patients were included in our study [296 females (88.62%) and 38 males (11.38)]. We categorized the patients into three groups:

- **Group 1**: patients receiving JAK Inhibitor (baricitinib) included 150 patients and represented 44.91% of the study population.
- **Group 2**: patients receiving TNF inhibitors (etanercept 33.33%, golimumab 35.72%, and adalimumab 30.95%) included 84 patients and represented 25.15% of the study population.
- **Group 3**: patients receiving cDMARDs included 100 patients and represented 29.94% of the study population.

### Demographic and clinical data of the patients at enrollment to the study

One hundred fifty patients received the JAK Inhibitor baricitinib, 129(86%) were females, and 21 (14%) were males. The mean age of this group was 44.24 ± 12.12 years, and the mean disease duration was 3.39 ± 1.72 years. Eighty-four patients received TNF Inhibitors, including etanercept, golimumab, and adalimumab. 75 (89.3%) were females, and 9 (10.7%) were males. The mean age of this group was 42.39 ± 11.07 years, and the mean disease duration was 3.58 ± 1.82 years. One hundred patients received cDMARDs, 92(92%) were females, and 8 (8%) were males. The mean age of this group was 45.9 ± 11.04 years, and the mean disease duration was 3.49 ± 1.74 years. Gender, age, and disease duration were comparable in the three groups (Table 1).

**Table 1:** Demographic and clinical data of the patients at enrollment

Parameters		Baricitinib (n = 150)	TNF Inhibitors (n = 84)	cDMARDs (n = 100)	P value
Gender	*Females*	129 (86%)	75 (89.3%)	92 (92%)	0.334
	*Males*	21 (14%)	9 (10.7%)	8(8%)	
Age (*Mean*** ± ***SD)*		44.24 ± 12.12	42.39 ± 11.07	45.9 ± 11.04	0.123
Disease duration (years)		3.39 ± 1.72	3.58 ± 1.82	3.49 ± 1.74	0.741
Morning Stiffness no.(%)		137(91.3%)	81(96.4%)	92(92%)	0.327
Arthralgia no. (%)		150(100%)	84(100%)	98(98%)	0.095
Arthritis no. (%)		150 (100%)	84(100%)	99(99%)	0.309
Extra-articular no. (%)		37 (24.7%)	23(27.4%)	25 (25.0%)	0.515
TJC ( mean ± SD)		12.26 ± 6.13	13.24 ± 6.89	11.05 ± 4.67	0.044*
SJC ( mean ± SD)		7.4 ± 6.09	8.05 ± 6.4	6.23 ± 2.45	0.063
VAS ( mean ± SD)		6.6 ± 1.87	6.3 ± 1.68	6.82 ± 1.62	0.146
DAS28 ( mean ± SD)		6.52 ± 1.07	6.31 ± 1.19	6.49 ± 1.06	0.326
CDAI ( mean ± SD)		22.76 ± 12.06	24.13 ± 10.53	24.96 ± 7.75	0.255
HAQ-DI ( mean ± SD)		1.95 ± 0.57	2.08 ± 0.5	2.05 ± 0.59	0.182
Larsen Score ( mean ± SD)		77.37 ± 21.13	76.7 ± 20.39	73.51 ± 18.45	0.316
ESR ( mean ± SD)		71.18 ± 25.64	61.77 ± 24.54	64.12 ± 21.7	0.008**
CRP ( mean ± SD)		34.9 ± 32.28	29.8 ± 23.1	28.24 ± 15.98	0.109
RF positivity no. (%)		133(88.7%)	70(83.3%)	81(81%)	0.296
ACPA positvity no. (%)		107(71.3)	68(81%)	79(79%)	0.368

Chi-Square, or one-way ANOVA test, was used wherever suitable

* The difference is significant at p < 0.05, ** the difference is significant at p < 0.01

TJC: Tender joint count. SJC: Swollen joint count. VAS: Visual analog scale

DAS28: Disease activity score for 28 joints. CDAI: Clinical disease activity index

HAQ-DI: Health Assessment Questionnaire Disability Index. RF.: Rheumatoid factor

ACPA: Anticitrullinated protein antibodies

Regarding the clinical manifestations of the participants at the time of enrollment to the study, the most common manifestations were arthritis and arthralgia, which were present in all the patients in the baricitinib and TNF Inhibitors groups, and 99% and 98% of the patients in the cDMARDs group respectively. The second most common manifestation was morning stiffness, which was present in 91.3%, 96.4%, and 92% of the patients receiving baricitinib, TNF Inhibitors, and cDMARDs, respectively. Extra-articular manifestations were found in 24.7%, 27.4%, and 25% of the patients receiving baricitinib, TNF Inhibitors, and cDMARDs, respectively. The three groups showed no significant differences regarding the clinical manifestations. Also, no significant differences were detected between the 3 groups regarding SJC, VAS, DAS28, CDAI, HAQ-DI, and modified Larsen score. But, there was a significant difference regarding TJC (P = 0.044). Regarding the laboratory findings, the ESR was higher in the baricitinib group (p = 0008) with no significant difference regarding CRP, RF, and ACPA positivity (Table 1).

### Therapeutic data of the patients

**Fig. 2:**
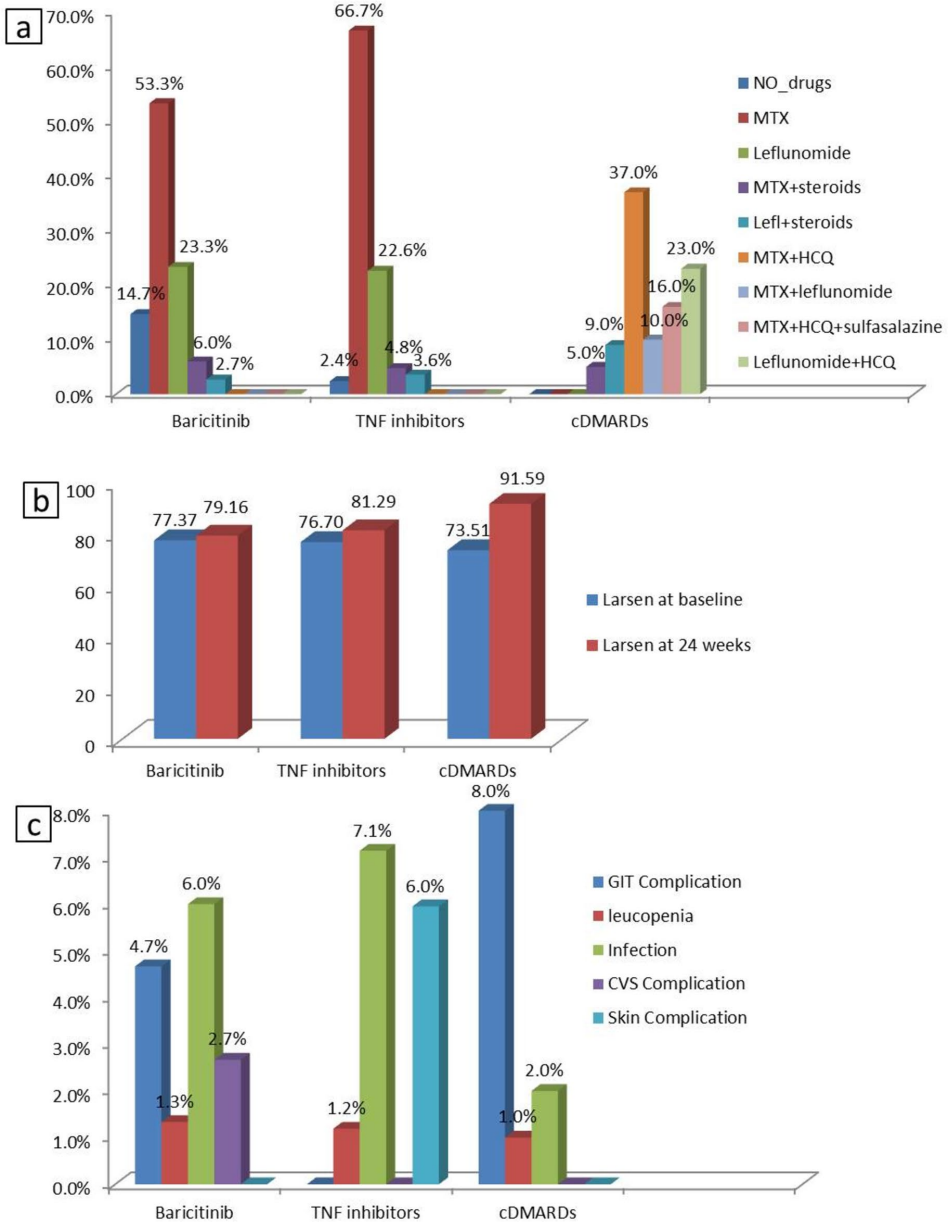
**a**) Therapeutic lines used by the patients in the three groups, **b**) Larsen score at baseline and twenty-four weeks, **c**) The observed side effects of the medications during the follow-up period

For the baricitinib group, 80 (53.33%) of the patients were receiving methotrexate, 35 (23.3%) were receiving Leflunamide. And 9 (6%) were receiving methotrexate and low-dose corticosteroids, 4 (2.7%) were receiving leflunomide and low-dose corticosteroids as background therapy and 22 (14.67%) were using baricitinib as a monotherapy. Regarding the TNF Inhibitors group, 56 (66.67%) of the patients were receiving methotrexate, 19 (22.62%) were receiving leflunomide, 4 (4.76) were receiving methotrexate and low-dose corticosteroids, 3 (3.57%) were receiving leflunomide and low-dose corticosteroids as background therapy and 2 (2.38%) were using TNF inhibitors as a monotherapy. For patients in the cDMARDs group, the most commonly used combination was methotrexate and hydroxychloroquine in 37% of the patients, followed by leflunamide and hydroxychloroquine in 23% of the patients, Methotrexate, hydroxychloroquine, and sulfasalazine in 16% of the patients, Methotrexate and Leflunamide in 10% of patients, Leflunomide and low-dose corticosteroids in 9% of the patients, methotrexate and low-dose corticosteroids in 5% of the patients (Fig. 2a).

### The outcome measures for patients receiving baricitinib compared with those receiving TNF Inhibitors

**At baseline:** On comparing patients receiving baricitinib with those receiving TNF Inhibitors, no significant difference was detected between the two groups regarding all measured parameters (Table 2).

**Table 2:** Comparison of the outcome measures between baricitinib and TNF Inhibitors groups at baseline, 12 and 24 weeks

Parameters	Baricitinib (n = 150)	TNF Inhibitors (n = 84)	P value
At baseline			
TJC ( mean ± SD)	12.26 ± 6.13	13.24 ± 6.89	0.264
SJC ( mean ± SD)	7.4 ± 6.09	8.05 ± 6.4	0.449
VAS ( mean ± SD)	6.6 ± 1.87	6.31 ± 1.69	0.239
DAS28 ( mean ± SD)	6.52 ± 1.07	6.3 ± 1.19	0.155
CDAI ( mean ± SD)	22.76 ± 12.06	24.13 ± 10.53	0.384
HAQ-DI ( mean ± SD)	1.95 ± 0.57	2.08 ± 0.5	0.084
Larsen Score (Mean ± SD)	77.37 ± 21.13	76.7 ± 20.39	0.816
At week 12			
TJC (Mean ± SD)	5.46 ± 5.02	4.71 ± 4.26	0.252
STC ( mean ± SD)	2.8 ± 4.14	2 ± 2.27	0.103
DAS 28 ( mean ± SD)	4.47 ± 1.39	4.38 ± 1.37	0.626
CDAI ( mean ± SD)	16.65 ± 12.88	14.76 ± 11.89	0.269
VAS ( mean ± SD)	3.27 ± 2.07	3.68 ± 2.37	0.175
HAQ-DI ( mean ± SD)	1.24 ± 0.63	1.35 ± 0.62	0.179
ACR 20 no. (%)	54 (36%)	27 (32%)	0.552
ACR 50 no. (%)	45 (30%)	35(41%)	0.071
ACR 70 no. (%)	32 (21.3%)	7(8.3%)	0.01*
At week 24			
TJC ( mean ± SD)	3.33 ± 3.79	3.74 ± 3.61	0.427
STC ( mean ± SD)	1.85 ± 2.48	1.5 ± 2.22	0.280
DAS28 ( mean ± SD)	3.99 ± 1.16	4.09 ± 1.37	0.569
CDAI ( mean ± SD)	10.82 ± 9.92	11.08 ± 9.89	0.849
VAS (Mean ± SD)	2.03 ± 1.56	2.36 ± 1.84	0.141
HAQ-DI ( mean ± SD)	0.95 ± 0.62	1 ± 0.63	0.519
Larsen score (Mean ± SD)	79.16 ± 21.62	81.28 ± 21.09	0.467
ACR20 no. (%)	12 (8%)	11 (13.1%)	0.209
ACR50 no. (%)	40(26.7%)	27(32.1%)	0.374
ACR70 no. (%)	92(61.3%)	44 (52.4%)	0.183

Independent samples t-test or Chi-Square test was used wherever possible

*The difference is significant at p < 0.05

TJC: Tender joint count. SJC: Swollen joint count. VAS: Visual analog scale

DAS28: Disease activity score for 28 joints. CDAI: Clinical disease activity index

HAQ-DI: Health Assessment Questionnaire Disability Index

ACR20, ACR50, ACR70: American College of Rheumatology 20%, 50%, and 70% response criteria

**At week 12**: The two groups were comparable regarding all measured parameters except for the ACR70 response, which was higher in the baricitinib group than in the TNF inhibitors group (p = 0.01) (Table 2).

**At week 24:** The two groups were comparable in all measured parameters (Table 2).

### The outcome measures for patients receiving baricitinib compared with those receiving cDMARDs

**At baseline:** When we compared the baricitinib group with the cDMARDs group, we found no significant difference in all measured parameters (Table 3).

**Table 3:** Comparison of the outcome measures between baricitinib and cDMARDs groups at baseline, 12, and 24 weeks

Parameters	Baricitinib (n = 150)	cDMARDs (n = 100)	P value
At baseline			
TJC ( mean ± SD)	12.26 ± 6.12	11.05 ± 4.67	0.095
SJC ( mean ± SD)	7.4 ± 6.09	6.23 ± 2.45	0.068
VAS ( mean ± SD)	6.6 ± 1.87	6.82 ± 1.62	0.338
DAS28 ( mean ± SD)	6.52 ± 1.07	6.49 ± 1.06	0.838
CDAI ( mean ± SD)	22.76 ± 12.06	24.96 ± 7.75	0.108
HAQ-DI ( mean ± SD)	1.95 ± 0.57	2.04 ± 0.59	0.197
Larsen Score ( mean ± SD)	77.37 ± 21.13	73.51 ± 18.45	0.139
At week 12			
TJC ( mean ± SD)	5.46 ± 5.02	8.14 ± 4.26	< 0.001***
SJC ( mean ± SD)	2.8 ± 4.15	4.47 ± 3.39	0.001**
VAS ( mean ± SD)	3.27 ± 2.07	4.03 ± 1.47	0.002**
DAS28 (Mean ± SD)	4.47 ± 1.39	5.75 ± 1.35	< 0.001***
CDAI ( mean ± SD)	16.65 ± 12.88	20.67 ± 9.35	0.008**
HAQ-DI ( mean ± SD)	1.24 ± 0.63	1.41 ± 0.46	0.019*
ACR 20 no. (%)	54 (36%)	24 (24%)	0.045*
ACR 50 no. (%)	45 (30%)	18 (18%)	0.032*
ACR 70 no. (%)	32 (21.3%)	4 (4%)	< 0.001***
At week 24			
TJC ( mean ± SD)	3.33 ± 3.8	7.6 ± 4.09	< 0.001***
SJC ( mean ± SD)	1.85 ± 2.48	3.93 ± 3.06	< 0.001***
VAS ( mean ± SD)	2.03 ± 1.56	3.81 ± 1.76	< 0.001***
DAS28 (Mean ± SD)	3.99 ± 1.16	5.65 ± 1.35	< 0.001***
CDAI (Mean ± SD)	10.83 ± 9.92	19.63 ± 10.49	< 0.001***
HAQ-DI ( mean ± SD)	0.95 ± 0.62	1.37 ± 0.59	< 0.001***
Larsen Score (Mean ± SD)	79.16 ± 21.62	91.49 ± 17.42	< 0.001***
ACR 20 no. (%)	12 (8%)	12 (12%)	0.293
ACR 50 no. (%)	40 (26.7%)	16 (16%)	0.048*
ACR 70 no. (%)	92 (61.3%)	25 (25%)	< 0.001***

Independent samples t-test or Chi-Square test was used wherever possible

* The difference is significant at p < 0.05, ** the difference is significant at p < 0.01, and *** the difference is significant at p < 0.001

TJC: Tender joint count. SJC: Swollen joint count.VAS: Visual analog scale

DAS28: Disease activity score for 28 joints. CDAI: Clinical disease activity index

HAQ-DI: Health Assessment Questionnaire Disability Index

ACR20, ACR50, ACR70: American College of Rheumatology 20%, 50%, and 70% response criteria

**At week 12:** There were significant improvements in all outcome measures in the baricitinib group compared with the cDMARDs group, including TJC (p < 0.001), SJC (P = 0.001), VAS (p = 0.002), DAS28 (p < 0.001), CDAI (p = 0.008), HAQ-DI (p = 0.019). In addition, more patients achieved ACR20, ACR50, and ACR70 responses in the baricitinib group than in the cDMARDs group (p = 0.045), (p = 0.032), and (p < 0.001) respectively (Table 3).

**At week 24:** An improvement in all the outcome measures was observed in the baricitinib group compared with the cDMARDs group, including TJC (p < 0.001), SJC(p < 0.001), VAS (p < 0.001), DAS28 (p < 0.001), CDAI (p < 0.001), HAQ-DI (p < 0.001), and Larsen score (p < 0.001). In addition, more patients achieved ACR50 and ACR70 responses in the baricitinib group than in the cDMARDs group (p = 0.048) and (p < 0.001), respectively (Table 3).

### Analysis of the outcome measures in the baricitinib group at different time points

In patients receiving baricitinib, when we compared the outcome measures at baseline with those at 12 and 24 weeks, we found a significant improvement regarding TJC, SJC, VAS, DAS28, CDAI, and HAQ-DI (p < 0.001). Also, a significant improvement was observed at week 24 compared to week 12 regarding TJC, VAS, DAS28, CDAI, HAQ-DI (p < 0.001), and SJC (p = 0.001). In addition, more patients achieved ACR70 response at week 24 compared with week 12 (p < 0.001). On using the modified Larsen score for measuring structural joint damage, we found a significantly increased Larsen score at week 24 compared to baseline (p < 0.001) (Table 4). Despite the increased Larsen score at week 24 compared with the baseline, the radiological progression in patients receiving baricitinib was comparable to that of patients receiving TNF inhibitors and less manifested than that of patients receiving cDMARDs (Fig. 2b).

**Table 4:** Comparison of the outcome measures at different time points in the baricitinib group

parameters	mean ± SD	P value	parameters	mean ± SD, NO(%)	P value
TJC baseline	12.26 ± 6.13	< 0.001***	DAS28 (12 weeks)	4.47 ± 1.39	< 0.001***
TJC (12 weeks)	5.46 ± 5.02		DAS28 (24 weeks)	3.99 ± 1.16	
TJC baseline	12.26 ± 6.13	< 0.001***	CDAI (baseline)	22.76 ± 12.06	< 0.001***
TJC (24 weeks)	3.33 ± 3.79		CDAI (12 weeks)	16.65 ± 12.88	
TJC (12 weeks)	5.46 ± 5.02	< 0.001***	CDAI (baseline)	22.76 ± 12.06	< 0.001***
TJC (24 weeks)	3.33 ± 3.79		CDAI (24 weeks)	10.83 ± 9.92	
SJC baseline	7.4 ± 6.09	< 0.001***	CDAI (12 weeks)	16.65 ± 12.88	< 0.001***
SJC(12 weeks)	2.8 ± 4.15		CDAI (24 weeks)	10.83 ± 9.92	
SJC baseline	7.4 ± 6.09	< 0.001***	HAQ-DI (baseline)	1.95 ± 0.57	< 0.001***
SJC (24 weeks)	1.85 ± 2.48		HAQ-DI (12 weeks)	1.24 ± 0.63	
SJC (12 weeks)	2.8 ± 4.15	0.001**	HAQ-DI (baseline)	1.95 ± 0.57	< 0.001***
SJC (24 weeks)	1.85 ± 2.48		HAQ-DI (24 weeks)	0.95 ± 0.62	
VAS (baseline)	6.60 ± 1.87	< 0.001***	HAQ-DI (12 weeks)	1.24 ± 0.63	< 0.001***
VAS (12 weeks)	3.27 ± 2.07		HAQ-DI (24 weeks)	0.95 ± 0.62	
VAS (baseline)	6.6 ± 1.87	< 0.001***	Larsen (baseline)	77.37 ± 21.13	< 0.001***
VAS (24 weeks)	2.03 ± 1.56		Larsen (24 weeks)	79.16 ± 21.62	
VAS (12 weeks)	3.27 ± 2.07	< 0.001***	ACR20 (12 weeks)	54(36%)	< 0.001***
VAS (24 weeks)	2.03 ± 1.56		ACR20 (24 weeks)	12(8%)	
DAS28(baseline)	6.52 ± 1.072	< 0.001***	ACR50 (12 weeks)	45(30%)	0.644
DAS28 (12 weeks)	4.47 ± 1.39		ACR50 (24 weeks)	40(26.7%)	
DAS28 (baseline)	6.52 ± 1.07	< 0.001***	ACR70 (12 weeks)	32(21.3%)	< 0.001***
DAS28 (24 weeks)	3.99 ± 1.16		ACR70 (24 weeks)	92(61.3%)	

Paired samples t-test, or McNemar test, was used wherever possible

** The difference is significant at p < 0.01, and *** the difference is significant at p < 0.001

TJC: Tender joint count. SJC: Swollen joint count. VAS: Visual analog scale

DAS28: Disease activity score for 28 joints. CDAI: Clinical disease activity index

HAQ-DI: Health Assessment Questionnaire Disability Index

ACR20, ACR50, ACR70: American College of Rheumatology 20%, 50%, and 70% response criteria

### The observed side effects of the medications during the follow-up period

In the baricitinib group, infection was the most common side effect (6.66%), followed by GIT complications (4.66%), CVS complications (2.66%), and leucopenia (1.33%). For The TNF Inhibitors group, the most common side effects were infection (7.14%), skin complications (5.95%), and leucopenia (1.19%). Regarding The cDMARDs group, the most common side effects were GIT complications (8%), infection (2%), and leucopenia (1%) (Fig. 2c).

## Discussion

Rheumatoid arthritis is a progressive, potentially deforming type of autoimmune inflammatory arthritis [25]. Despite symmetrical polyarthritis being the hallmark, extra-articular manifestations and long-term complications are not uncommon [4, 8, 26]. For Decades, methotrexate monotherapy and a combination of other synthetic, conventional DMARDs have been the standard first-line therapy for RA [27]. Unfortunately, about two-thirds of patients may fail treatment with cDMARDs and remain with poor or no disease control [28]. The introduction of biological DMARDs, especially TNF-α inhibitors, represented a new hope for RA patients, especially those with resistant or refractory disease [9, 11]. Although many patients could achieve reasonable disease control on bDMARDs, about one-third may still fail treatment with bDMARDs [29]. Moreover, biological therapies are known for their high cost and exclusive parenteral route of administration. This has raised the need for newer agents with lower costs, more feasible routes of administration, and, most importantly, comparable efficacy.

JAK Inhibitors have been the newest agents to be added to RA treatment regimens [5]. These new agents are characterized as a small molecule-targeted therapy, less costly, and can be administered orally. They have also proved their efficacy compared to the existing bDMARDs, making them a promising option for treating refractory RA. Previous studies highlighted their effectiveness and safety profile [30–32]**.** Our study aimed to assess the efficacy of the JAK inhibitor (baricitinib) in patients with no or poor disease control despite using cDMARDs and to compare it with TNF inhibitors and cDMARDs.

According to our findings, in patients treated with baricitinib, TJC, SJC, DAS28 score, CDAI score, VAS scores, and HAQ-DI all showed a significant improvement at week 12 compared to baseline (p < 0.001). In addition, 36% of the patients achieved ACR 20 response, 30% achieved ACR 50 response, and 21.3% achieved ACR 70 response. This was in agreement with the published results of [33], which demonstrated a significant improvement of RA disease activity measurements observed after two weeks of therapy, with a continued effect up to week 12 in patients treated with baricitinib. A substantial improvement in the ACR 20 response rate and the HAQ-DI score was reported by [31] at week 12 of treatment with baricitinib compared with placebo. At week 24, we observed a sustained and continuous improvement in all outcome measures, including TJC, SJC, DAS28 score, CDAI score, VAS scores, and HAQ-DI, compared to baseline and week 12 (p < 0.001). In addition, there was a significant improvement in ACR70 responses at 24 weeks compared to 12 weeks (p < 0.001). [34] and [35] also proved the efficacy of baricitinib on all the studied outcome measures throughout the first 24 weeks of treatment. [36] agreed with our findings regarding significantly better HAQ-DI and VAS scores in patients treated with baricitinib compared to placebo. [37] showed a significant improvement in ACR20, ACR50, and ACR70, as well as DAS28 and CDAI scores at week 24 of baricitinib treatment, as compared to placebo.

When we compared the patients receiving baricitinib with those receiving TNF inhibitors, no significant difference was observed at week 12, except for the ACR 70 response rate, which was significantly higher in the baricitinib group (p = 0.01). At week 24, no statistically significant difference was observed between both groups regarding all measured parameters, including TJC, SJC, VAS, DAS28, CDAI score, HAQ-DI, Larsen score, ACR20, ACR50, and ACR70 response rates. Our findings were In agreement with [15] and [38]**,** who found that baricitinib at least has the same, or maybe better response rates than TNF inhibitors and explained this by the inhibitory effect of this new agent on the JAK/STAT pathway involved in the synthesis of many mediators responsible for the inflammatory process in RA, and proved by many studies [39, 40]. A recent study showed that the outcome of RA patients on baricitinib was comparable to or better than TNF inhibitors, and suggested that RA patients who failed to respond to cDMARDs, are recommended to start baricitinib which is a feasible alternative to TNF inhibitors [41].

On comparing the baricitinib with the cDMARDs group: At week 12, baricitinib showed significantly better results than cDMARDs regarding all the outcome measures, including TJC (p < 0.001), SJC (p = 0.001), VAS (p = 0.002), DAS28 (p < 0.001), CDAI (p = 0.008), HAQ-DI (p = 0.019), ACR20 (p = 0.045), ACR50 (p = 0.032), and ACR 70 (p < 0.001) response rates. This effect continued throughout the study and up to week 24, with significantly better outcome measures in patients receiving baricitinib than those receiving cDMARDs, including TJC (p < 0.001), SJC (p < 0.001), VAS (p < 0.001), DAS28(p < 0.001), CDAI (p < 0.001), HAQ-DI (p < 0.001), ACR50 (p = 0.048), and ACR 70 response rates (p < 0.001) [42]. reported the superiority of baricitinib as monotherapy or in combination with cDMARDs over methotrexate upon evaluating all the outcome measures included in their study. In addition, at week 24, we observed a significant superiority of the baricitinib over the cDMARDs regarding the delay of radiographic disease progression, as measured by the Modified Larsen Score (p < 0.001). In agreement with our findings [43] and [44] also proved the superiority of baricitinib regarding inhibition of radiographic disease progression**.** This can be further explained by the findings of [45], who reported that the inactivation of both JAK1 and JAK2 produced by baricitinib resulted in the inhibition of the process of subchondral bony erosions and, subsequently, the radiographic disease progression in RA patients.

Evaluation of the side effects emerging during the study period showed the highest incidence of adverse effects with both baricitinib and TNF inhibitors compared to cDMARDs, which had the lowest incidence and severity of side effects. The most common side effects of baricitinib were Infection, GIT, and CVS complications. In agreement with our findings, [46] noted that infection was the most common adverse effect of JAK inhibitors. The incidence of infection in patients receiving baricitinib was comparable to those receiving TNF inhibitors. In agreement with our findings, [13] and [38] found nearly similar infection rates in patients using JAK Inhibitors and TNF inhibitors. Other observed side effects include leucopenia, which appeared in 1.33% of the JAK inhibitors group and 1.19% of the TNF Inhibitors group. In agreement with our findings, [47] and [38] noticed leucocytic count reductions in patients receiving JAK Inhibitors, and [48] reported leucocytic count reductions in patients receiving TNF inhibitors. In addition, the TNF inhibitors group showed other adverse effects, including cutaneous complications ranging from simple injection site reactions to multiple skin abscesses. Our findings agree with [49]. According to our results, cDMARDs had the least adverse effects; the most common was GIT upset, followed by urinary tract infection. In agreement with our findings, [38] and [50] found that cDMARDs had a better safety profile than both bDMARDs and tsDMARDs.

## Conclusion

Baricitinib is an effective drug for treating RA refractory to cDMARDs, improving disease activity measures and functional status, and reducing the progression of structural joint damage. It has a comparable efficacy and safety profile to TNF Inhibitors. Multicenter studies are recommended to support our results.

## Data Availability

The current study's data are available from the corresponding author upon reasonable request.

## Declarations

### Study Limitations

It is a single-center, small sample-size study, limited by biological and targeted therapy costs.

## References

[1] Littlejohn EA, Monrad SU (2018) Early diagnosis and treatment of rheumatoid arthritis. Prim Care 45:237–255. 10.1016/j.pop.2018.02.010 29759122 10.1016/j.pop.2018.02.010

[2] Cojocaru M, Cojocaru IM, Silosi I, Vrabie CD, Tanasescu R (2010) Extra-articular manifestations in rheumatoid arthritis. Maedica (Bucur). 5: 286–91. https://pmc.ncbi.nlm.nih.gov/articles/PMC3152850/PMC3152850 21977172

[3] Carbone F, Bonaventura A, Liberale L, Paolino S, Torre F, Dallegri F, Cutolo M et al (2020) Atherosclerosis in Rheumatoid Arthritis: Promoters and Opponents. Clin Rev Allergy Immunol 58:1–14. 10.1007/s12016-018-8714-z 30259381 10.1007/s12016-018-8714-z

[4] Cush JJ (2021) Rheumatoid Arthritis: Early Diagnosis and Treatment. Med Clin North Am 105:355–365. 10.1016/j.mcna.2020.10.006 33589108 10.1016/j.mcna.2020.10.006

[5] England BR, Tiong BK, Bergman MJ, Curtis JR, Kazi S, Mikuls TR, Michaud K et al (2019) 2019 Update of the American College of Rheumatology Recommended Rheumatoid Arthritis Disease Activity Measures. Arthritis Care Res 71:1540–1555. 10.1002/acr.24042 10.1002/acr.24042 PMC6884664 31709779

[6] Fraenkel L, Bathon JM, England BR, St Clair EW, Arayssi T, Carandang K, Akl EA et al (2021) 2021 American College of Rheumatology Guideline for the Treatment of Rheumatoid Arthritis. Arthritis Rheumatol (Hoboken, N.J.) 73:1108–1123. 10.1002/art.41752 10.1002/art.41752 34101376

[7] Burmester GR, Pope JE (2017) Novel treatment strategies in rheumatoid arthritis. Lancet (London, England) 389:2338–2348. 10.1016/S0140-6736(17)31491-5 28612748 10.1016/S0140-6736(17)31491-5

[8] Aletaha D, Smolen JS (2018) Diagnosis and Management of Rheumatoid Arthritis: A Review. JAMA 320:1360–1372. 10.1001/jama.2018.13103 30285183 10.1001/jama.2018.13103

[9] Shah P, Siddique A, Thakkar A, Gharat S, Godad A, Kale P, Doshi G (2022) An update on novel therapeutic intervention in Rheumatoid arthritis. Int Immunopharmacol 109:108794. https://jamanetwork.com/journals/jama/article-abstract/2705192 10.1016/j.intimp.2022.108794 35504203

[10] Petrović-Rackov L (2006) Evaluation of the degree of clinical rheumatoid arthritis activity based on the concentrations of cytokines TNF-alpha, IL-12, IL-15, and IL-18 in serum and synovial fluid. Vojnosanit Pregl 63:21–26. 10.2298/vsp0601021p 16471244 10.2298/vsp0601021p

[11] Visvanathan S, Rahman MU, Keystone E, Genovese M, Klareskog L, Hsia E, Wagner C et al (2010) Association of serum markers with improvement in clinical response measures after treatment with golimumab in patients with active rheumatoid arthritis despite receiving methotrexate: results from the GO-FORWARD study. Arthritis Res Ther 12:R211. 10.1186/ar3188 21083889 10.1186/ar3188 PMC3046519

[12] Emery P, Breedveld F, van der Heijde D, Ferraccioli G, Dougados M, Robertson D, et al Etanercept in Early Rheumatoid Arthritis Trial G, (2010) Two-year clinical and radiographic results with combination etanercept-methotrexate therapy versus monotherapy in early rheumatoid arthritis: a two-year, double-blind, randomized study. Arthritis Rheumatism 62: 674-682. 10.1002/art.27268. 10.1002/art.27268 20187135

[13] Harrington R, Al Nokhatha SA, Conway R (2020) JAK Inhibitors in Rheumatoid Arthritis: An Evidence-Based Review on the Emerging Clinical Data. J Inflamm Res 13:519–531. 10.2147/JIR.S219586 32982367 10.2147/JIR.S219586 PMC7500842

[14] Hu L, Liu R, Zhang L (2022) Advance in bone destruction participated by JAK/STAT in rheumatoid arthritis and therapeutic effect of JAK/STAT inhibitors. Int Immunopharmacol 111:109095. https://www.sciencedirect.com/science/article/abs/pii/S1567576922005793 10.1016/j.intimp.2022.109095 35926270

[15] Barbulescu A, Askling J, Chatzidionysiou K, Forsblad-d’Elia H, Kastbom A, Lindström U, Frisell T et al (2022) Effectiveness of baricitinib and tofacitinib compared with bDMARDs in RA: results from a cohort study using nationwide Swedish register data. Rheumatology (Oxford) 61:3952–3962. 10.1093/rheumatology/keac068 35134119 10.1093/rheumatology/keac068 PMC9536798

[16] Aletaha D, Neogi T, Silman AJ, Funovits J, Felson DT, Bingham CO III, Cohen MD et al (2010) 2010 rheumatoid arthritis classification criteria: an American College of Rheumatology/European League Against Rheumatism collaborative initiative. Arthritis Rheumatism. 62:2569–2581. 10.1002/art.27584 20872595 10.1002/art.27584

[17] Lauper K, Iudici M, Mongin D, Bergstra SA, Choquette D, Codreanu C, Elkayam O et al (2022) Effectiveness of TNF-inhibitors, abatacept, IL6-inhibitors and JAK-inhibitors in 31 846 patients with rheumatoid arthritis in 19 registers from the ‘JAK-pot’collaboration. Ann Rheum Dis 81:1358–1366 35705376 10.1136/annrheumdis-2022-222586 PMC9484385

[18] Delgado DA, Lambert BS, Boutris N, McCulloch PC, Robbins AB, Moreno MR, Harris JD (2018) Validation of digital visual analog scale pain scoring with a traditional paper-based visual analog scale in adults. JAAOS Global Research & Reviews 2:e088. 10.5435/JAAOSGlobal-D-17-00088 30211382 10.5435/JAAOSGlobal-D-17-00088 PMC6132313

[19] Barczyńska TA, Dura M, Blumfield E, Węgierska M, Żuchowski P, Wilińska-Jankowska A, Jeka S (2015) DAS28 score vs. ultrasound examination for assessment of rheumatoid arthritis disease activity: comparison and discussion of pros and cons. Reumatologia/Rheumatology 53:213–218. 10.5114/reum.2015.53999 27407250 10.5114/reum.2015.53999 PMC4847288

[20] Dhaon P, Das SK, Srivastava R, Dhakad U (2018) Performances of Clinical Disease Activity Index (CDAI) and Simplified Disease Activity Index (SDAI) appear to be better than the gold standard Disease Assessment Score (DAS-28-CRP) to assess rheumatoid arthritis patients. Int J Rheum Dis 21:1933–1939. 10.1111/1756-185X.13110 28608433 10.1111/1756-185X.13110

[21] Maska L, Anderson J, Michaud K (2011) Measures of functional status and quality of life in rheumatoid arthritis: Health Assessment Questionnaire Disability Index (HAQ), Modified Health Assessment Questionnaire (MHAQ), Multidimensional Health Assessment Questionnaire (MDHAQ), Health Assessment Questionnaire II (HAQ-II), Improved Health Assessment Questionnaire (Improved HAQ), and Rheumatoid Arthritis Quality of Life (RAQoL). Arthritis Care Res 63(Suppl 11):S4-13. 10.1002/acr.20620 10.1002/acr.20620 22588760

[22] Felson DT, Anderson JJ, Boers M, Bombardier C, Furst D, Goldsmith C, Strand V et al (1995) American College of Rheumatology. Preliminary definition of improvement in rheumatoid arthritis. Arthritis Rheum 38:727–735. 10.1002/art.1780380602 7779114 10.1002/art.1780380602

[23] Felson DT, Anderson JJ, Lange ML, Wells G, LaValley MP (1998) Should improvement in rheumatoid arthritis clinical trials be defined as fifty percent or seventy percent improvement in core set measures, rather than twenty percent? Arthritis Rheum 41:1564–1570. 10.1002/1529-0131(199809)41:9%3c1564::AID-ART6%3e3.0.CO;2-M 9751088 10.1002/1529-0131(199809)41:9<1564::AID-ART6>3.0.CO;2-M

[24] Rau R, Herborn G (1995) A modified version of Larsen's scoring method to assess radiologic changes in rheumatoid arthritis. J Rheumatol 22:1976–1982. https://www.researchgate.net/publication/14216212 8992004

[25] Zhong X, Feng W, Liu L, Liu Q, Xu Q, Liu M, Lin C et al (2024) Periplogenin inhibits pathologic synovial proliferation and infiltration in rheumatoid arthritis by regulating the JAK2/3-STAT3 pathway. Int Immunopharmacol 128:111487. 10.1016/j.intimp.2024.111487 38183911 10.1016/j.intimp.2024.111487

[26] Radu AF, Bungau SG (2021) Management of Rheumatoid Arthritis: An Overview. Cells. 10. 10.3390/cells10112857. 10.3390/cells10112857 PMC8616326 34831081

[27] Favalli EG, Biggioggero M, Meroni PL (2014) Methotrexate for the treatment of rheumatoid arthritis in the biologic era: still an “anchor” drug? Autoimmun Rev 13:1102–1108. 10.1016/j.autrev.2014.08.026 25172238 10.1016/j.autrev.2014.08.026

[28] Sethi MK, O’Dell JR (2015) Combination conventional DMARDs compared to biologicals: what is the evidence? Curr Opin Rheumatol 27:183–188. 10.1097/BOR.0000000000000153 25603037 10.1097/BOR.0000000000000153

[29] Smolen JS, Aletaha D, Koeller M, Weisman MH, Emery P (2007) New therapies for treatment of rheumatoid arthritis. Lancet (London, England) 370:1861–1874. 10.1016/S0140-6736(07)60784-3 17570481 10.1016/S0140-6736(07)60784-3

[30] Cai W, Tong R, Sun Y, Yao Y, Zhang J (2024) Comparative efficacy of five approved Janus kinase inhibitors as monotherapy and combination therapy in patients with moderate-to-severe active rheumatoid arthritis: a systematic review and network meta-analysis of randomized controlled trials. Front Pharmacol 15:1387585. 10.3389/fphar.2024.1387585 38725657 10.3389/fphar.2024.1387585 PMC11080655

[31] Genovese MC, Kremer J, Zamani O, Ludivico C, Krogulec M, Xie L, Smolen JS et al (2016) Baricitinib in Patients with Refractory Rheumatoid Arthritis. N Engl J Med 374:1243–1252. 10.1056/NEJMoa1507247 27028914 10.1056/NEJMoa1507247

[32] Liao X, Huo W, Zeng W, Qin F, Dong F, Wei W, Lei L (2023) Efficacy and safety of different Janus kinase inhibitors combined with methotrexate for the treatment of rheumatoid arthritis: a single-center randomized trial. Advances in Rheumatology 63:50. 10.1186/s42358-023-00331-1 37845778 10.1186/s42358-023-00331-1

[33] Tanaka Y, Emoto K, Cai Z, Aoki T, Schlichting D, Rooney T, Macias W (2016) Efficacy and Safety of Baricitinib in Japanese Patients with Active Rheumatoid Arthritis Receiving Background Methotrexate Therapy: A 12-week, Double-blind, Randomized Placebo-controlled Study. J Rheumatol 43:504–511. 10.3899/jrheum.150613 26834213 10.3899/jrheum.150613

[34] Dougados M, Dvd H, Chen Y-C, Greenwald M, Drescher E, Liu J, Emery P et al (2015) LB0001 Baricitinib, an Oral Janus Kinase (JAK)1/JAK2 Inhibitor, in Patients with Active Rheumatoid Arthritis (RA) and An Inadequate Response to CDMARD Therapy: Results of the Phase 3 RA-Build Study. Ann Rheum Dis 74:79–79. 10.1136/annrheumdis-2015-eular.6539

[35] Genovese MC, Kremer J, Zamani O, Ludivico C, Krogulec M, Xie L, Smolen JS et al (2015) OP0029 Baricitinib, An Oral Janus Kinase (JAK)1/JAK2 Inhibitor, in Patients with Active Rheumatoid Arthritis (RA) and an Inadequate Response to TNF Inhibitors: Results of the Phase 3 RA-Beacon Study. Ann Rheum Dis 74:75–76. 10.1136/annrheumdis-2015-eular.1427

[36] Emery P, Blanco R, Maldonado Cocco J, Chen YC, Gaich CL, DeLozier AM, Dougados M et al (2017) Patient-reported outcomes from a phase III study of baricitinib in patients with conventional synthetic DMARD-refractory rheumatoid arthritis. RMD Open 3:e000410. 10.1136/rmdopen-2016-000410 28405473 10.1136/rmdopen-2016-000410 PMC5372156

[37] Keystone EC, Taylor PC, Drescher E, Schlichting DE, Beattie SD, Berclaz P-Y, Genovese MC et al (2015) Safety and efficacy of baricitinib at 24 weeks in patients with rheumatoid arthritis who have had an inadequate response to methotrexate. Ann Rheum Dis 74:333–340. 10.1136/annrheumdis-2014-206478 25431052 10.1136/annrheumdis-2014-206478 PMC4316868

[38] Taylor PC, Keystone EC, van der Heijde D, Weinblatt ME, Del Carmen ML, Reyes Gonzaga J, Tanaka Y et al (2017) Baricitinib versus Placebo or Adalimumab in Rheumatoid Arthritis. N Engl J Med 376:652–662. 10.1056/NEJMoa1608345 28199814 10.1056/NEJMoa1608345

[39] Serra López-Matencio JM, Morell Baladrón A, Castañeda S (2019) JAK-STAT inhibitors for the treatment of immunomediated diseases. Med Clin (Barc) 152:353–360. 10.1016/j.medcli.2018.10.020 30527218 10.1016/j.medcli.2018.10.020

[40] Kisseleva T, Bhattacharya S, Braunstein J, Schindler CW (2002) Signaling through the JAK/STAT pathway, recent advances and future challenges. Gene 285:1–24. 10.1016/s0378-1119(02)00398-0 12039028 10.1016/s0378-1119(02)00398-0

[41] van de Laar CJ, Voshaar MAO, Ten Klooster P, Tedjo DI, Bos R, Jansen T, Kroot E-J et al (2024) PERFECTRA: a pragmatic, multicentre, real-life study comparing treat-to-target strategies with baricitinib versus TNF inhibitors in patients with active rheumatoid arthritis after failure on csDMARDs. RMD Open 10:e004291 38816210 10.1136/rmdopen-2024-004291 PMC11328659

[42] Fleischmann R, Schiff M, van der Heijde D, Ramos-Remus C, Spindler A, Stanislav M, Takeuchi T et al (2017) Baricitinib, Methotrexate, or Combination in Patients With Rheumatoid Arthritis and No or Limited Prior Disease-Modifying Antirheumatic Drug Treatment. Arthritis Rheumatol 69:506–517. 10.1002/art.39953 27723271 10.1002/art.39953 PMC5347954

[43] van der Heijde D, Schiff M, Tanaka Y, Xie L, Meszaros G, Ishii T, Emery P et al (2019) Low rates of radiographic progression of structural joint damage over 2 years of baricitinib treatment in patients with rheumatoid arthritis. RMD Open 5:e000898. 10.1136/rmdopen-2019-000898 31168413 10.1136/rmdopen-2019-000898 PMC6525612

[44] Emery P, Durez P, Hueber AJ, de la Torre I, Larsson E, Holzkämper T, Tanaka Y (2021) Baricitinib inhibits structural joint damage progression in patients with rheumatoid arthritis-a comprehensive review. Arthritis Res Ther 23:3. 10.1186/s13075-020-02379-6 33397481 10.1186/s13075-020-02379-6 PMC7784289

[45] Murakami K, Kobayashi Y, Uehara S, Suzuki T, Koide M, Yamashita T, Nakamura Y et al (2017) A Jak1/2 inhibitor, baricitinib, inhibits osteoclastogenesis by suppressing RANKL expression in osteoblasts in vitro. PLoS ONE 12:e0181126. 10.1371/journal.pone.0181126 28708884 10.1371/journal.pone.0181126 PMC5510865

[46] Winthrop KL (2017) The emerging safety profile of JAK inhibitors in rheumatic disease. Nat Rev Rheumatol 13:234–243. 10.1038/nrrheum.2017.23 28250461 10.1038/nrrheum.2017.23

[47] Huang F, Luo Z-C (2018) Risk of Adverse Drug Events Observed with Baricitinib 2 mg Versus Baricitinib 4 mg Once Daily for the Treatment of Rheumatoid Arthritis: A Systematic Review and Meta-Analysis of Randomized Controlled Trials. BioDrugs: Clinical Immunotherapeutics. Biopharmaceuticals and Gene Therapy 32:415–423. 10.1007/s40259-018-0304-3 10.1007/s40259-018-0304-3 30203252

[48] He B, Li Y, Luo W-w, Cheng X, Xiang H-r, Zhang Q-z, Peng W-x et al (2022) The Risk of Adverse Effects of TNF-α Inhibitors in Patients With Rheumatoid Arthritis: A Network Meta-Analysis. Front Immunol 13:814429. 10.3389/fimmu.2022.814429 35250992 10.3389/fimmu.2022.814429 PMC8888889

[49] Hernández MV, Sanmartí R, Cañete JD, Descalzo MA, Alsina M, Carmona L, Gomez-Reino JJ (2013) Cutaneous adverse events during treatment of chronic inflammatory rheumatic conditions with tumor necrosis factor antagonists: study using the Spanish registry of adverse events of biological therapies in rheumatic diseases. Arthritis Care Res (Hoboken) 65:2024–2031. 10.1002/acr.22096 23926075 10.1002/acr.22096

[50] Wei JC-C, Tsou H-K, Leong P-Y, Chen C-Y, Huang J-X (2020) Head-to-Head Comparison of Etanercept vs. Adalimumab in the Treatment of Ankylosing Spondylitis: An Open-Label Randomized Controlled Crossover Clinical Trial. Front Med 7:566160. 10.3389/fmed.2020.566160 10.3389/fmed.2020.566160 PMC7662505 33195311

